# SpectralMAE: Spectral Masked Autoencoder for Hyperspectral Remote Sensing Image Reconstruction

**DOI:** 10.3390/s23073728

**Published:** 2023-04-04

**Authors:** Lingxuan Zhu, Jiaji Wu, Wang Biao, Yi Liao, Dandan Gu

**Affiliations:** 1 School of Electronic Engineering, Xidian University, Xi’an 710071, China; wujj@mail.xidian.edu.cn (J.W.);; 2 National Key Laboratory of Scattering and Radiation, Shanghai 200438, China

**Keywords:** spectral reconstruction, hyperspectral imaging, masked autoencoder, self-supervised learning, transformer

## Abstract

Accurate hyperspectral remote sensing information is essential for feature identification and detection. Nevertheless, the hyperspectral imaging mechanism poses challenges in balancing the trade-off between spatial and spectral resolution. Hardware improvements are cost-intensive and depend on strict environmental conditions and extra equipment. Recent spectral imaging methods have attempted to directly reconstruct hyperspectral information from widely available multispectral images. However, fixed mapping approaches used in previous spectral reconstruction models limit their reconstruction quality and generalizability, especially dealing with missing or contaminated bands. Moreover, data-hungry issues plague increasingly complex data-driven spectral reconstruction methods. This paper proposes SpectralMAE, a novel spectral reconstruction model that can take arbitrary combinations of bands as input and improve the utilization of data sources. In contrast to previous spectral reconstruction techniques, SpectralMAE explores the application of a self-supervised learning paradigm and proposes a masked autoencoder architecture for spectral dimensions. To further enhance the performance for specific sensor inputs, we propose a training strategy by combining random masking pre-training and fixed masking fine-tuning. Empirical evaluations on five remote sensing datasets demonstrate that SpectralMAE outperforms state-of-the-art methods in both qualitative and quantitative metrics.

## 1. Introduction

**Figure 1:**
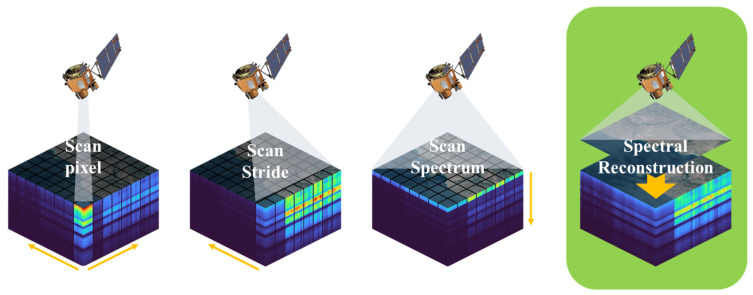
Comparison of sensor scan-based remote sensing hyperspectral imaging mechanisms and spectral reconstruction methods.

Hyperspectral imaging (HSI) provides higher spectral resolution with contiguous and narrow bands, enabling a wealth of information to be captured for applications such as semantic segmentation [1,2], scene classification [3,4,5], object detection [6,7] and target tracking [8,9]. Nonetheless, due to the limitations of the spectral imaging mechanism, the trade-off between spatial and spectral resolution is a challenge in remote sensing. The increased number of spectral bands results in an information bottleneck due to the space-bandwidth product of the detector arrays. As shown in Figure 1, conventional hyperspectral imaging techniques, such as push-broom detectors and digital array detectors, rely on scanning spatial or spectral dimensions to generate the three-dimensional data cube. This imaging process is time-consuming and limited to static objects. In time-sensitive applications and situations where there is relative motion between objects and the environment, the introduction of time-series hyperspectral imaging introduces the possibility of spatial misalignment and subsequent distortion of recorded spectra. To address these limitations, there is a need for new and innovative approaches that can enhance spectral resolution without sacrificing spatial resolution.

The pursuit of high spatial resolution, spectral resolution and imaging speed in hardware is cost-intensive and relies on rigorous environmental conditions and extra equipment. An attractive option for solving the addressed problem is to use lower-cost, more available multi-spectral images (MSI) or RGB images to directly reconstruct hyperspectral information, widely known as the spectral reconstruction technique (SR). Spectral reconstruction techniques provide a cost-effective solution to the hardware limitations in remote sensing. By leveraging a wider range of data sources, SR methods allow for more efficient utilization of sensors, leading to the improved overall utilization of remote sensing satellites.

The mainstream research of hyperspectral image reconstruction includes spectral super-resolution [10,11,12,13] and computational spectral reconstruction from RGB to HSI [14,15,16,17,18]. In general, recovering hyperspectral information is an ill-posed inverse problem. The spectral reconstruction approaches are usually categorized into two kinds [19]: prior-based and data-driven methods.

- Prior-based methods

Traditional hyperspectral reconstruction employs prior knowledge of HSIs as a regularizer to solve such a seriously ill-posed problem. The representative prior-based methods include dictionary learning [20,21,22,23,24], Gaussian Process [25] and manifold mapping [26]. Dictionary learning methods express the spectral signal with multiple combinations of base spectra. The accuracy and abundance of the dictionary representation determine the reconstruction quality of the spectral signal. The subsequent research work enhanced the dictionary representation by combining a priori knowledge such as local Euclidean linearity [20], spatial structure similarity [23], texture [22], spatial context [24], etc. Manifold learning methods characterize hyperspectral images as unique low-dimensional manifolds. It solves the spectral reconstruction problem to a many-to-many mapping problem and simplifies the mapping complexity by recovering low-dimensional embedding. Gaussian Process methods use a priori knowledge of spectral physics and spatial structure similarity. Gaussian Process methods model the spectral characteristics of the material categories contained in the image. In training, it divides the images into different clusters by algorithms such as K-means and learns spectral response functions in the different clusters. In test, the hyperspectral image is reconstructed using the characterization parameters and the Gaussian Process.

These hand-crafted priors have limitations and often need to reflect all the characteristics of the data. In the case of an unknown camera sensitivity function, the reconstruction performance and accuracy will be reduced. These severely restrict the performance and generalization ability of the prior-based methods mentioned above.

- Data-driven methods

Pioneering works have proved that the performance of data-driven priors exceeds that of hand-crafted priors. Embracing more and more datasets, data-driven methods acquire powerful feature representation ability and good adaptability. Various deep learning networks have been proposed to improve reconstruction accuracy, from simple network models to more advanced deep learning networks using a variety of techniques. These data-driven methods, which primarily use supervised learning, include CNN-based methods [14,27], GAN-based methods [16,28,29] and Attention-based methods [18,30,31]. CNN-based methods have strong nonlinear characterization capability and are widely used in SR. Compared to RGB images, CNN methods for hyperspectral images tend to use higher dimensional convolution kernels to adapt to the spectral dimension. Given the recent advances in deep learning, various variants of the SR method based on CNN models have been derived. U-Net models [32] combine multi-scale information through sampling transformation. Dense Networks [17] and Residual Networks [33] avoid the vanishing of the gradient and richer image details. Multi-branch networks [34] fuse multi-path signal flow to obtain diverse features. The above approaches can be applied as a general framework for subsequent SR network design. The GAN-based methods consist of a generator and a discriminator. The reconstruction of images is enhanced by adversarial learning between two networks. The Attention-based methods, which introduced patterns from the NLP, can be more capable of modeling at long-range distances. Compared to CNNs, the Attention mechanism can focus differently on the spatial and spectral dimensions.

However, the limitations of the above-mentioned model should also be considered. One of the primary drawbacks is that the mapping relationship between the target and input spectra is fixed and unidirectional, rendering the model invalid in the event of changes to the imaging device or input spectral bands. Additionally, the spectral reconstruction performance can be significantly impacted by missing or disturbed input bands resulting from interference or attacks. Thus, there is a pressing need for more robust spectral reconstruction techniques capable of handling such scenarios and providing reliable results.

Moreover, data-hungry issues plague increasingly complex neural network models. Hyperspectral datasets much smaller than visible images limit the accuracy of SR models. A common way is to pre-train the model on another larger dataset. In recent years, self-supervised learning (SSL) [35] has emerged as an attractive approach for visual pre-training and has been shown to outperform its supervised counterpart. This is an active area of research and holds promising potential for improving the accuracy of hyperspectral image reconstruction models.

With the goal of improving the adaptability of SR models to the inputs of different spectral sensors and enhancing the utilization of remote sensing data, we propose a novel approach, the masked autoencoder-based spectral image reconstruction model (SpectralMAE). SpectralMAE reconceptualizes the spectral reconstruction problem based on the mask-then-predict strategy. This model leverages the power of autoencoder and masked modeling to provide a more robust and flexible solution for hyperspectral image reconstruction.

**Figure 2:**
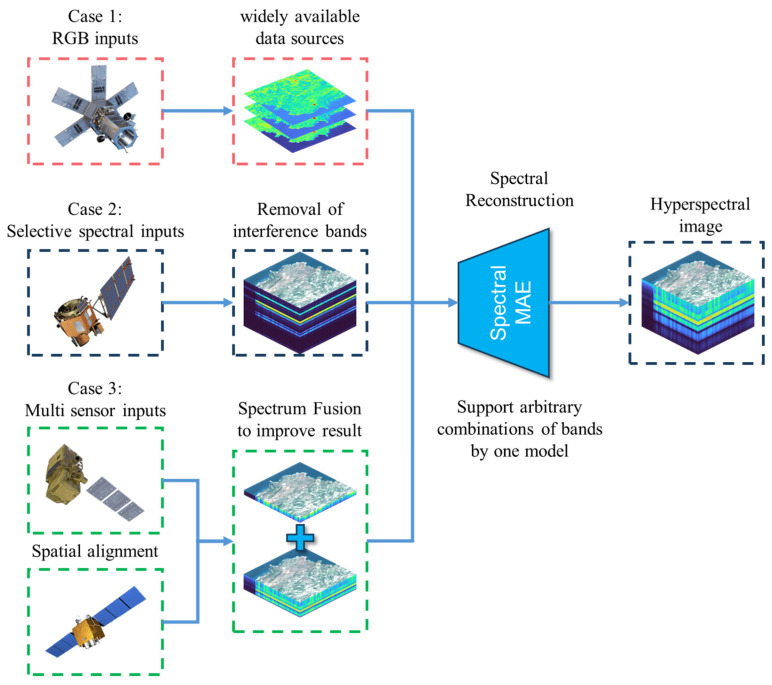
Typical application scenarios for SpectralMAE.

Figure 2 illustrates the capabilities of SpectralMAE in addressing three typical problems with a single model. First, it can generate remote sensing hyperspectral images directly from RGB images. Second, it can selectively input band combinations when dealing with partially damaged or incomplete band images. Third, it can fuse and reconstruct data from multiple sensors with spatial registration using spectral position encoding.

The contributions of our proposed SpectralMAE method can be summarized in three points:

1. Improved potential adaptability to various spectral sensors: The SpectralMAE model offers a more robust and flexible solution for hyperspectral image reconstruction, as it supports arbitrary combinations of spectral bands as inputs, including inputs from different numbers of multi-spectral data sources, RGB images and even a mixture of two sensors. Furthermore, compared to spectral super-resolution, SpectralMAE performs better in predicting spectral curves in non-overlapping spectral regions.
2. Maximizing the utilization of hyperspectral remote sensing data: The SpectralMAE model adopts a new paradigm of self-supervised learning for the spectral reconstruction problem. The model incorporates a mechanism of random masking during the training process. This mechanism leads to an exponential increase in the number of input combinations that the model must consider during training, resulting in a deeper understanding of the association between local and global features.
3. Improved reconstruction accuracy: The SpectralMAE model employs a two-stage training process, with pre-training using random masks followed by fine-tuning with fixed masks. By incorporating a positional encoding strategy for the spectral dimension and a transformer network architecture, the model is able to effectively capture and differentiate spectral information, even in the presence of masked positions. The proposed SpectralMAE model can outperform existing supervised learning methods on several remote sensing hyperspectral image datasets and can surpass 95% of the random masking rate in the spectral dimension.

## 2. Methodology

### 2.1. Overview of the Method

**Figure 3:**
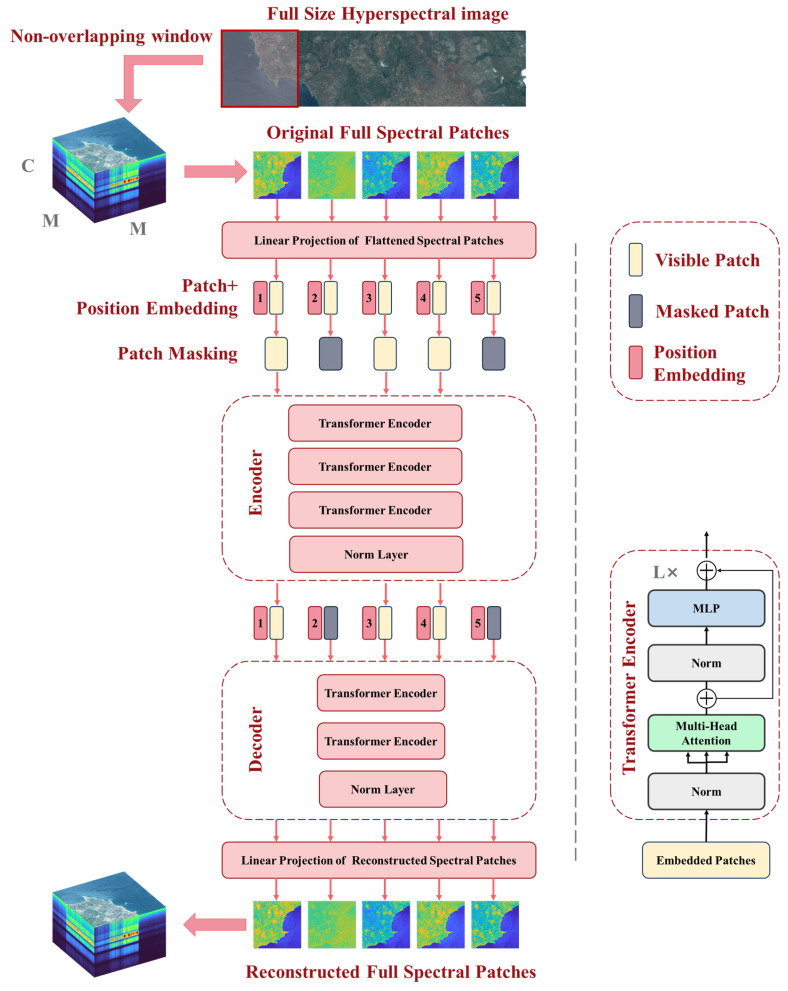
The architecture of Spectral Masked Autoencoder, where C represents the spectral dimension, M represents the Patch size and L indicates the number of Transformer encoders. The numbers 1 to 5 in position embedding indicate the relative positions of the spectral bands, and the position coordinates in the actual scene range from 1 to the full spectral dimension.

SpectralMAE models the spectral image reconstruction problem as predicting masked patches from the visible ones. As shown in Figure 3, the input spectral images are processed as visible patches, while the bands to be predicted are processed as masked patches. The visible patches are first fed into the encoder, and then the decoder uses the outputs of the encoder as well as the masked patches to reconstruct the complete spectral image. SpectralMAE requires performing masking operations in the spectral dimension, in contrast to imageMAE [36], which applies random masking operations in the spatial dimension.

### 2.2. Architecture of Spectral Masked Autoencoder

As depicted in Figure 3, SpectralMAE utilizes an asymmetric autoencoder comprising an encoder and a decoder. Both the encoder and decoder employ the vanilla Vision Transformer (ViT) backbone [37], which can handle masked inputs and position embedding directly. The encoder is limited to representing latent features using only the visible patches, while the decoder takes full spectral patches to accomplish the reconstruction task with lighter transformer blocks.

#### 2.2.1. Patch Embedding

The hyperspectral image $I\in {\mathbb{R}}^{H\times W\times C}$ is reshaped into a sequence of equal-sized non-overlapping 2D patches $I_{Spectral}$, where the total number of patches is $T=\left(H\times W\times C\right)/\left(M\times M\right)$. The spectral patch is defined as $I_{Spectral}\left(i,j,k\right)\in {\mathbb{R}}^{M\times M}$, where *M* represents the window width, *i* and *j* are the row and column of the non-overlapping window and k stands for the position of the patch in the spectral dimension. Each patch of a channel can be considered as a two-dimensional grayscale image. The encoder receives a sequence of patches $x_{p}\in {\mathbb{R}}^{rC\times M\times M}$ from different channels at the same location as input, where r represents the mask ratio.

Patches are mapped into one-dimensional feature vectors $z_{0}$ through a linear projection layer, which adapts the standard input format of the Transformer.

(1) $$z_{0}=\left[x_{p}^{1}E;x_{p}^{2}E;x_{p}^{3}E;\cdots ;x_{p}^{C}E\right]$$

where $E\in {\mathbb{R}}^{M\times M\times D}$ is the convolutional kernel and D is the dimensionality of the mapped feature vector.

#### 2.2.2. Position Embedding

Position embeddings [37] were added to all spectral tokens and provide information about their relative location in the spectrum, especially for the mask tokens.

(2) $$z_{1}=z_{0}+\left[p_{1};p_{2};p_{3};\cdots ;p_{C}\right]$$

SpectralMAE employs a 1D absolute position embedding for the spectral coordinates of the patches.

(3) $$\left\{\begin{array}{c} p_{k,2n}=\sin\left({k/10,000}^{2n/D}\right)\\ p_{k,2n+1}=\cos\left({k/10,000}^{2n/D}\right) \end{array}\right.$$

where $p_{k,2n}$ and $p_{k,2n+1}$ are the n-th and n+1-th components of the position embedding at the spectral position k, respectively.

#### 2.2.3. Masking

After the position embeddings are added, the masking operation is performed. The positions of the randomly masked patches follow a uniform distribution, and the masking rate determines the number of patches to be masked.

First, a set of indexes of masked positions are generated based on random numbers. The encoder receives the visible flattened patch as input and performs the encoding calculation. Next, a zero-set token is inserted into the output of the encoder according to the indexes of the masked positions, thus forming the complete decoder input. Lastly, the input token of the decoder is added to the position embedding again.

#### 2.2.4. Autoencoding

The autoencoder follows an asymmetric encoder–decoder design and processes the embedding tokens via a series of visual Transformer encoder blocks [38]. As shown in Figure 3, the visual Transformer encoder block comprises a multi-head self-attention module (MSA) [39] and feedforward neural network (FFN), coupled with layer normalization (LN) and residual connection [35]. Layer normalization is used to make the input distribution more stable. Residual connections are applied after every block to avoid degradation of the model.

(4) $$\begin{array}{cc} z\prime_{l+1}=\mathrm{MSA}\left(\mathrm{LN}\left(z_{l}\right)\right)+z_{l} & l=1\ldots L \end{array}$$

(5) $$\begin{array}{cc} z_{l+1}=\mathrm{MLP}\left(\mathrm{LN}\left(z\prime_{l+1}\right)\right)+z\prime_{l+1} & l=1\ldots L \end{array}$$

where L stands for the number of self-attention modules, and MLP is the multilayer perceptron.

The decoder’s output is reshaped to form reconstructed patches, and the last layer of the decoder is a linear projection followed by a hyperbolic tangent function (tanh). Finally, the patches at different spatial locations were merged into reconstructed hyperspectral images.

#### 2.2.5. Window-Based Spectral-Wised MSA

**Figure 4:**
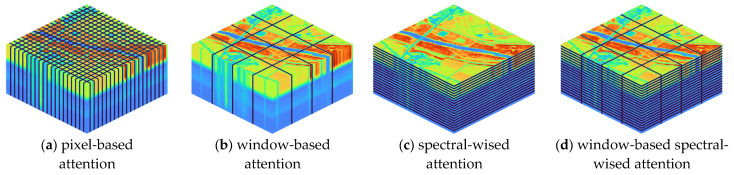
Four typical self-attention modules in the field of spectral reconstruction. The fourth one is used by SpectralMAE.

In recent years, researchers have proposed various attention modules, such as spatial attention [37], window-based attention [40], spectral-wise attention [18,31] and joint ones. As illustrated in Figure 4, pixel-based attention samples all pixels as key and query elements; window-based attention samples all tokens inside each window to calculate self-attention; and spectral-wise attention treats each channel as a token and calculates attention along the spectral dimension.

In contrast, our proposed SpectralMAE utilizes a novel mechanism called Window-based Spectral-wise Multi-head Attention (WS-MSA), which focuses on the correlation between spectral bands and learns the latent representation by interacting with all channels. WS-MSA calculates self-attention inside local spatial windows along the spectral dimension, allowing SpectralMAE to capture long-range relationships better. This results in improved performance methods and makes SpectralMAE an affordable approach for HSI reconstruction.

Suppose $X_{in}\in {\mathbb{R}}^{C\times D}$ as the input matrix of WS-MSA, where C denotes the number of channels. The scaled dot-product attention $A\in {\mathbb{R}}^{D\times D}$ is obtained by projection matrix query $Q\in {\mathbb{R}}^{C\times D}$, key $K\in {\mathbb{R}}^{C\times D}$, value $V\in {\mathbb{R}}^{C\times D}$.

(6) $$Q=X_{in}W^{Q},K=X_{in}W^{K},V=X_{in}W^{V}$$

where $W^{Q}\in {\mathbb{R}}^{D\times D}$, $W^{K}\in {\mathbb{R}}^{D\times D}$ and $W^{V}\in {\mathbb{R}}^{D\times D}$ are learnable parameters. WS-MSA calculates the self-attention for each $head_{i}$ and splits Q, K and V into N heads.

The dimension of each head is $d_{h}=D/N$. WS-MSA calculates the self-attention $A_{i}$ for each head and projects the concatenated outputs.

(7) $$A_{i}=\mathrm{softmax}\left(\frac{K_{i}^{T}Q_{i}}{\sqrt{D}}\right),head_{i}=V_{i}A_{i}$$

(8) $$\mathrm{WS}\text{-}\mathrm{MSA}=\left[head_{1};head_{2};head_{3};\cdots ;head_{N}\right]U$$

where $U\in {\mathbb{R}}^{D\times D}$ are learnable parameters. Subsequently, we reshape the attention maps and obtain the result $X_{out}\in {\mathbb{R}}^{D\times D}$.

In addition, the computation of patches for the position-specific window instead of inputting the complete image of a single channel can compress the feature vector dimension and make the characterization more powerful in the finite hidden dimension.

### 2.3. Training Strategy

**Figure 5:**
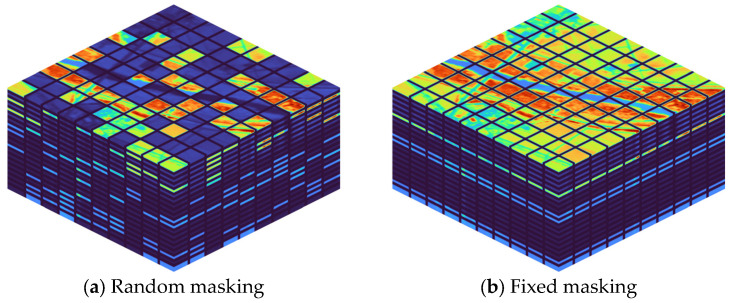
Schematic diagram of different training strategies applied to hyperspectral images. Highlighted areas are visible patches; dark areas are masked patches.

During training, there are two masking strategies used: random masking and fixed masking, as depicted in Figure 5. In the spectral dimension, the random masking strategy removes a certain percentage of input patches based on a uniform distribution. The fixed masking strategy, on the other hand, only masks the bands that need to be reconstructed.

Therefore, a pre-trained model is first constructed through self-supervised learning using the random masking strategy. This pre-trained model facilitates fast adaptation to various reconstruction tasks.

Additionally, it is observed that gradually increasing the masking rate makes the training more stable compared to setting it to the required masking rate at once. After the random masking reaches convergence, fine-tuning is performed by fixing the masking position. This step enables the model to achieve higher reconstruction accuracy than the pre-trained model.

### 2.4. Application Method

**Figure 6:**
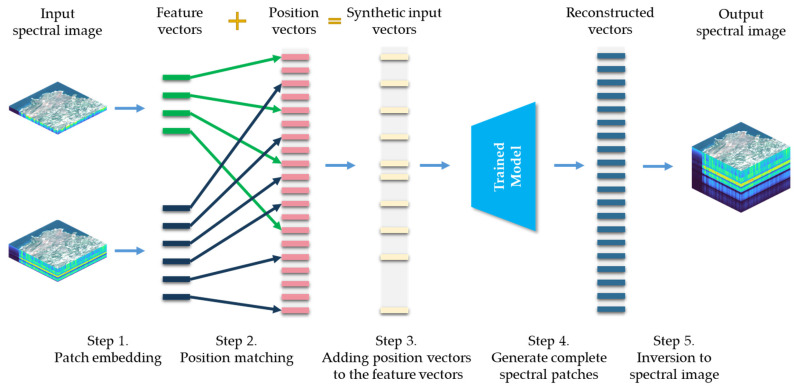
The specific steps of applying the SpectralMAE model, demonstrated using Case 3 in Figure 2 as an example. Two input spectral images with different spectral coverage from two sensors are fused and reconstructed to obtain an image with higher spectral resolution.

The key step in applying the trained SpectralMAE model to a specific problem is the position embedding. The specific steps for model application are illustrated in Figure 6, taking Case 3 in Figure 2 as an example. Suppose there are two sensors with different spectral coverage and their positionally registered spectral images need to be fused to reconstruct an image with higher spectral resolution. The first step is to transform the images of each band into feature vectors based on Equation (1). The second step is to determine the relative positions of each band in the complete spectral sequence and perform position encoding based on Equation (3). The third step is to add the position encoding vectors to the feature vectors, forming the input to the model. The fourth step is to output the reconstructed feature vectors based on the encoder and decoder of the trained model. The fifth step is to convert the reconstructed feature vectors into a spectral image using the linear projection layer and to synthesize the required complete spectral image.

### 2.5. Computational Cost

The computational complexity and storage requirement of the self-attention block has a quadratic dependence on the number of tokens [18]. The MSA module needs to calculate the Self-Attention module for each head.

(9) $$O\left(\mathrm{MSA}\right)=N_{\mathrm{head}}\cdot O\left(\mathrm{Self}\text{-}\mathrm{Attention}\right)=N_{\mathrm{head}}\left(n_{\mathrm{token}}{}^{2}d_{head}\right)$$

For hyperspectral images, we analyze the computational consumption of a single MSA block in the model. In SpectralMAE, the MSA module needs to be calculated $n=HW/M^{2}$ times, and $d_{head}=C/N$. The dimension of each head is $d_{head}=d_{h}=D/N$. Therefore, the computational complexity of SpectralMAE is

(10) $$O\left(\mathrm{SpectralMAE}\right)=n\cdot O\left(\mathrm{MSA}\right)=nN\left(C^{2}d_{h}\right)=\frac{HW}{M^{2}}\left(C^{2}D\right)$$

For the imageMAE model, if all spatial and spectral patches are considered at once, the number of tokens is $n_{\mathrm{token}}=\left(HW\right)/M^{2}$. Assuming that each patch has the same hidden dimension, the computational complexity of the imageMAE method is

(11) $$O\left(\mathrm{imageMAE}\right)=N\left[{\left(C\frac{HW}{M^{2}}\right)}^{2}d_{h}\right]={\left(\frac{HW}{M^{2}}\right)}^{2}\left(C^{2}D\right)$$

In contrast to imageMAE, whose computational cost is quadratic to the spatial size of the input, the computational cost of SpectralMAE is linear to the spatial size of the input. This means SpectralMAE significantly reduces imageMAE’s computational scale and training difficulty.

## 3. Experimental Results and Analysis

In this section, we compare our proposed method with 10 state-of-the-art methods, including 3 HSI reconstruction algorithms (MST++ [18], MST [31] and HDNet [12]); 3 super resolution methods (HSCNN+ [17], AWAN [15] and HRNet [41]); and 4 image restoration models (MPRNet [42], Restormer [43], HINet [44], and EDSR [45]). Among them, MST++ is the winning algorithm of NTIRE2022 [46]; HSCNN+ and AWAN are the winning algorithms of NTIRE in 2018 [47] and 2020 [48], respectively.

Table 1 presents an overview of above methods, categorized by their network architecture, along with a concise explanation of their basic idea and the year of publication. It is evident from the trend of development that attention and transformer structures have been extensively employed in spectral reconstruction models in recent years.

### 3.1. Dataset

We evaluate our proposed method on three multispectral remote sensing datasets (Chikusei [49], Washington DC Mall [50] and XiongAn [51]) and two hyperspectral remote sensing datasets (HyRANK [52] and GF2Hyper [53]).

The original Chikusei, Washington DC Mall and XiongAn airborne hyperspectral datasets have been down-sampled to the same number of channels as the Compact High Resolution Imaging Spectrometer (CHRIS), which contains 62 spectral channels ranging from 406 nm to 1003 nm. The processed Chikusei dataset comprises a total of 1016 training data. The processed Washington DC Mall dataset consists of 2424 training samples. The XiongAn dataset includes 1216 training samples. These three processed multispectral datasets were derived from the Sen2CHRIS dataset [53]. In the experiments, we used multispectral data from the dataset and extracted the corresponding visible wavelengths as input.

The HyRANK satellite hyperspectral data were obtained from the Hyperion sensor. After the band removal process, a total of 176 bands were derived. Five Hyperion surface reflectance datasets were obtained from the EarthExplorer platform. The GF2Hyper datasets include hyperspectral images captured by the Gaofen-1 and Hyperion sensors on the EO-1 satellite. In the dataset, 148 useful bands were selected through spectral sampling, and 1152 training samples were included. With these two datasets, we evaluated the model’s performance on satellite-based hyperspectral remote sensing images.

### 3.2. Implementation Details

During the training procedure, HSIs are spatially divided into non-overlapping patches as input data, which are then linearly rescaled to [0, 1]. The batch size is set to 32, and the input patch size of the model is 8 pixels. The Adam algorithm is used for parameter optimization. The learning rate is initialized as 0.00001, and the Cosine Annealing scheme is adopted for 500 epochs. The weight decay ratio is 0.0005, and the momentum is 0.9.

Additionally, gradient clipping is used to avoid the gradient explosion problem. In the first 200 epochs, random masking is performed in the spectral dimension of the data, and the masking rate is gradually increased from 75% to 90%. In the last 100 epochs, fixed masking is used, and the visible band is the input band of the reconstructed spectrum. The proposed SpectralMAE has been implemented on the Pytorch framework running in the Windows 10 environment and a single NVIDIA RTX A6000 GPU. In the experiment, the encoder of the SpectralMAE model contains 12 layers of the Transformer encoder network, where the embedding dimension of each network is 768, and the MSA module contains 12 attention heads. The lightweight decoder contains 8 layers of Transformer encoder networks, where the embedding dimension of each network is 512, and the MSA module contains 16 attention heads. The same number of training epochs and optimization methods are used for the comparative models, and the optimal results are obtained by adjusting the hyperparameters.

### 3.3. Qualitative Results

#### 3.3.1. Results of Hyperspectral Remote Sensing Image Datasets

- Results of the HyRANK dataset

**Figure 7:**
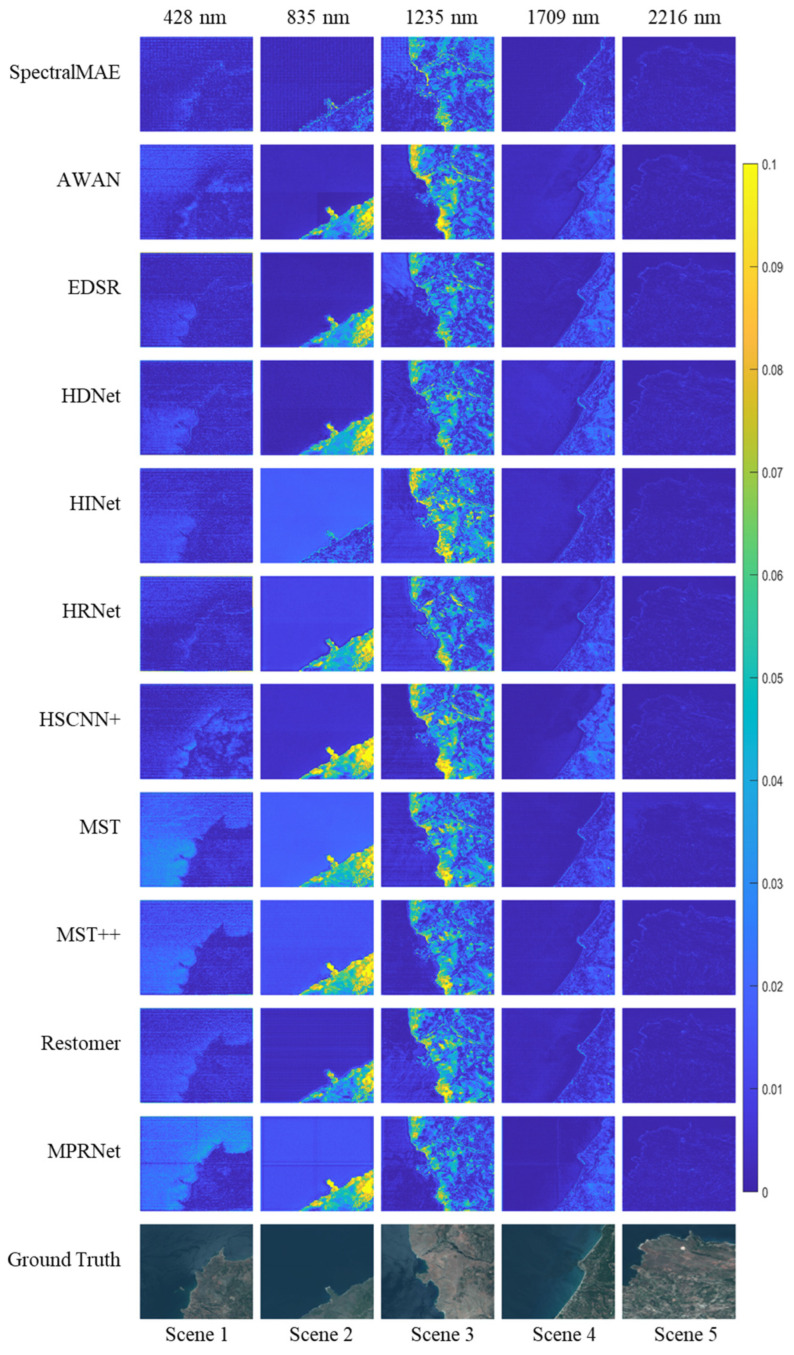
Visualization of the error maps between ground truth and reconstructed results on the HyRANK dataset with only three channel inputs.

We present the results of the HyRANK dataset with the proposed SpectralMAE and other state-of-the-art methods. Visual comparisons of error maps for five scenes in the HyRANK dataset are shown in Figure 7. All methods use the same three spectral channels as input to reconstruct a hyperspectral image with 176 channels.

Among the visualization results of the five bands, SpectralMAE shows less local variation than other compared methods. Most models exhibit different reconstruction errors for marine and terrestrial areas. The effects of topography and surface material types pose a more significant challenge to the spectral reconstruction of terrestrial regions. However, SpectralMAE still handles the differences between terrestrial and marine regions better, with the main errors originating from the over-region at the surface edge.

**Figure 8:**
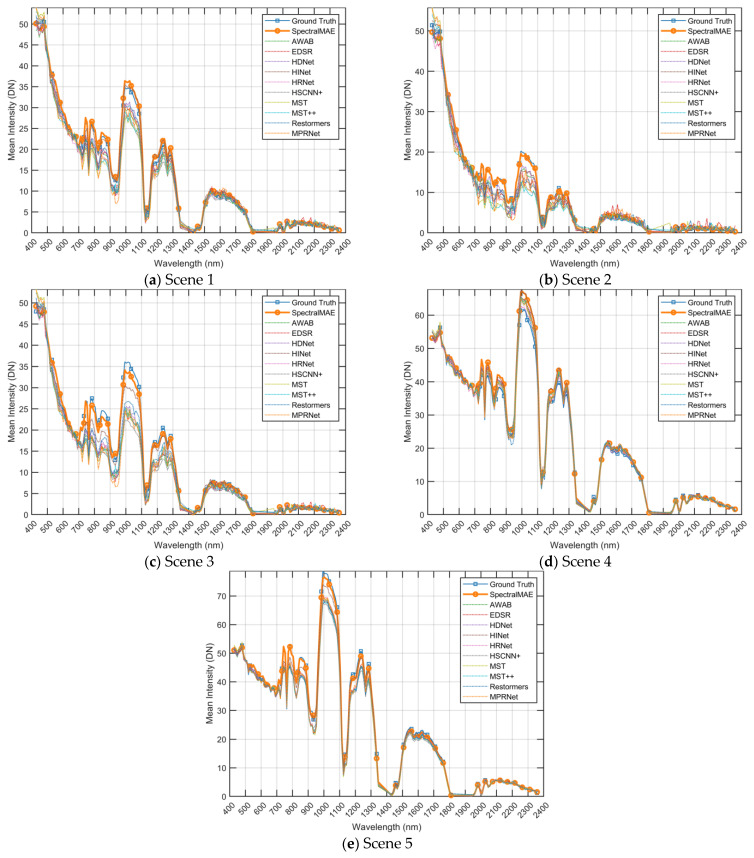
Reconstructed spectral curves comparisons of scenes shown in Figure 7 on the HyRANK dataset. The spectral curves were averaged in the spatial dimension of the images. DN is a digital number (uncalibrated) between 0 and 255.

To compare the performance in the spectral dimension, we present visualizations of spectral curves for the five scenes in Figure 7. As shown in the Figure 8, these curves represent the average value of each band in the image. While all methods are able to match the overall trend of the ground truth spectral curve, the reconstructed spectra of the comparison methods exhibit significant shifts in the wave peaks (e.g., at wavelengths between the 800 nm and 1000 nm and wavelengths between 1100 nm and 1300 nm). Additionally, these methods produce large fluctuations in the adjacent intervals. In contrast, the SpectralMAE method reconstructs spectral curves that closely match the ground truth. This is evident in the consistency of peaks and valleys of the spectra, as well as the smoothness of the excesses in the continuous region.

- Results of GF2Hyper Dataset

**Figure 9:**
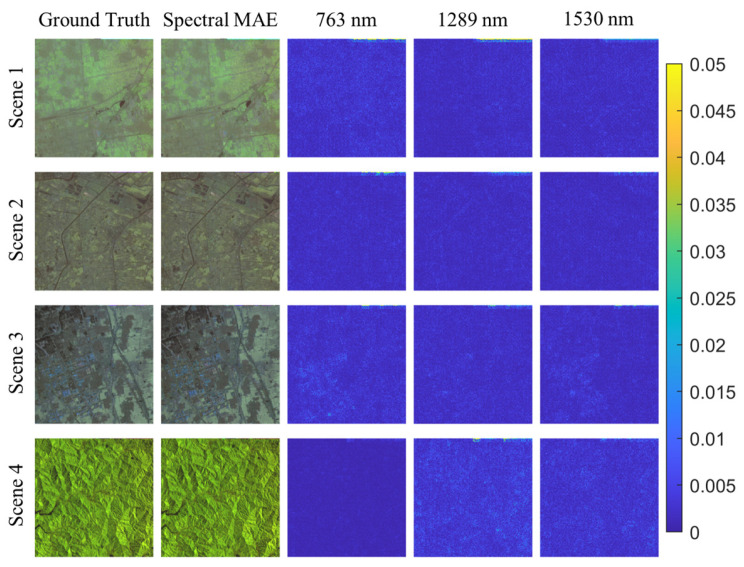
Visualization of the error maps between ground truth and the reconstructed results of SpectralMAE on the GF2Hyper dataset with only three channel inputs. The second column is the synthesized RGB image of the reconstructed spectral image.

Figure 9 illustrates the visual comparison results of the GF2Hyper dataset. The first two columns showcase pseudo-color images synthesized using wavelengths 763 nm, 1289 nm and 1530 nm. The last three columns depict error maps of the reconstructed image when compared to the ground-truth image. The error is primarily present in areas with solid undulations on the ground surface. Additionally, reconstruction errors can be observed at distorted locations on the edges of the original image.

#### 3.3.2. Results of Multispectral Remote Sensing Image Datasets

**Figure 10:**
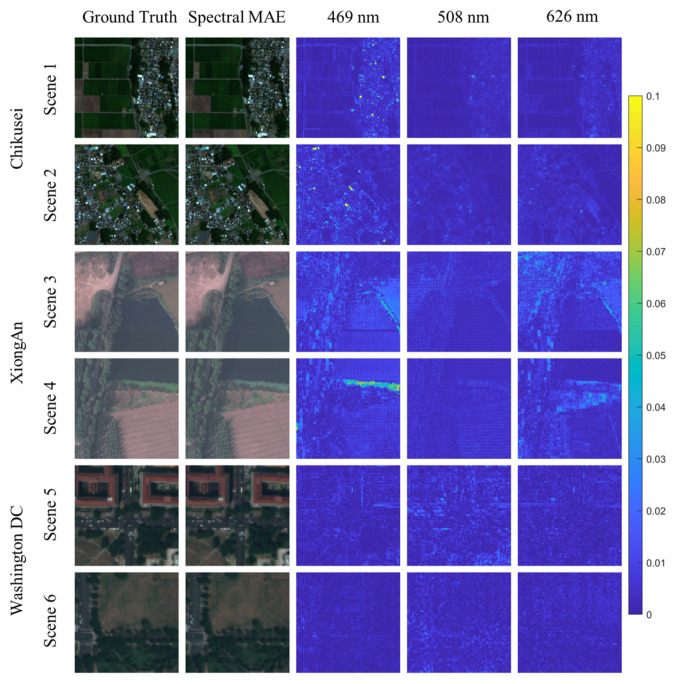
Visualization of the error maps between ground truth and the reconstructed results of SpectralMAE on Chikusei, XiongAn and Washington DC Mall datasets with only three channel inputs. The second column is the synthesized RGB image of the reconstructed spectral image.

Figure 10 visualizes the results of the spectral reconstruction of airborne multispectral images. The figure displays the error maps of the three visible bands, as well as their synthesized color images. For the Chikusei dataset, SpectralMAE demonstrates low errors for various types of land, with the majority of errors arising from buildings containing a small number of pixels in the images. For the XiongAn dataset, the errors primarily originate from the junction of lake and land surfaces, which occur less frequently in the scene. Additionally, the XiongAn dataset has a higher resolution of the ground surface, resulting in significant light and shadow effects, leading to additional errors due to the difference in brightness of the forest. In the Washington DC dataset, most areas are buildings and streets in the city, and most errors are found at the edges of buildings.

### 3.4. Quantitative Results

As for quantitative comparisons, we utilize four commonly used picture-quality indices (PQIs), including mean peak signal-to-noise ratio (mPSNR), mean structure similarity (mSSIM), root mean square error (RMSE) [54] and Spectral angle mapper (SAM) [55]. These PQIs enable us to evaluate the reconstructed HSIs in terms of their similarity to the ground truth. mPSNR and mSSIM are calculated on each 2D spatial image, which evaluates the similarity between the reconstructed HSIs and the ground truth based on MSE and structural consistency, respectively. SAM calculates the average angle between the spectrum vectors of the reconstructed HSIs and the ground truth.

Table 2 presents the average quantitative results on the HyRANK dataset. The HyRANK dataset contains five hyperspectral images, which we split into 128-pixel spatially-resolved patches for training and testing. We reserved 10% of the images as a test set and applied the same data augmentation techniques, such as image flipping, to all models. We optimized hyperparameters for comparison models and used the Adam algorithm with a batch size of 16, learning rate initialized to 0.00004 and Cosine Annealing scheme. After training, all models reached stable convergence.

Like many hyperspectral remote sensing datasets, the HyRANK dataset has a limited number of images. Training a model to reconstruct 172 spectral bands from this limited data is a challenging ill-posed problem. When facing higher-dimensional reconstruction problems with even smaller datasets, SR methods may exhibit prediction bias, as shown in Figure 7 and Figure 8. The proposed SpectralMAE outperforms all other competing supervised methods, not only in mPSNR, mSSIM and RMSE but also in SAM.

The prediction biases observed in other models may be attributed to their limited utilization of the data. In contrast, the SpectralMAE model achieved improved accuracy by implementing a “random mask-then-predict” strategy, which enabled the model to learn various combinations of mappings from the limited dataset.

Based on the principle of unordered permutation and combination, the number of mapping types on the spectral dimension increased to $C_{N}^{N\times \left(1-r\right)}$, where N is the number of spectral dimensions and r is the masking rate. Assume that mapping from RGB channels to 176-dimensional hyperspectral images constitutes one mapping type. Then the mapping types obtained by the self-supervised learning method of SpectralMAE contain $C_{176}^{3}=\frac{176\times 175\times 174}{3\times 2\times 1}=893,200$ types. Additionally, because the mask rate increases continuously during pre-training, the model eventually learns even more mapping types. Moreover, the fixed-masking strategy further improved the results.

In another aspect, SpectralMAE only considered the patch size in the spatial dimension, resulting in larger implicit parameters than models that read the entire image. By focusing the local windows during each batch, the original spatial structure of the image was disrupted, resulting in further data augmentation in the spatial dimension.

### 3.5. Ablation Study

#### 3.5.1. Quantitative Results of the Training Strategy

In this section, we conduct simulated experiments to show the effectiveness of our training strategy. Table 3 and Table 4 present the quantitative results of random masked hyperspectral image reconstruction at 75% and 90% masking rates, respectively. SpectralMAE achieves a higher mask ratio than imageMAE based on the assumption of high redundancy of spectral information. A higher masking rate means that the model acquires less information, making it more difficult to predict the unknown bands accurately. As a result, the results of a 90% masking rate are generally lower than those of a 75% masking rate. Due to the continuity of the spectrum, SpectralMAE can achieve a higher masking rate than imageMAE, which makes the reconstruction of spectral images possible.

Table 5 shows the reconstruction results under a fixed masking strategy. The input provided to the model comprises three images in the visible band. The model is a pre-trained model obtained by training with a 90% masking rate. Some datasets (e.g., Chikusei, XiongAn) have higher metrics than the random mask results, while some datasets (e.g., Washington DC Mall, GF2Hyper, HyRANK) have lower metrics than the random mask results. This is because the model trained using the random masking strategy is optimized to minimize training error for all input band combinations, including the average result between the best and worst combinations.

By utilizing the random mask model as a pre-trained model and fine-tuning it using the fixed mask strategy, we are able to obtain improved results, as evidenced by the results displayed in Table 6. These results highlight the effectiveness of our proposed training strategy, which effectively balances the trade-off between generalization and specialization, thus leading to improved performance.

#### 3.5.2. Spectral Curve Comparison Results of the Training Strategy

**Figure 11:**
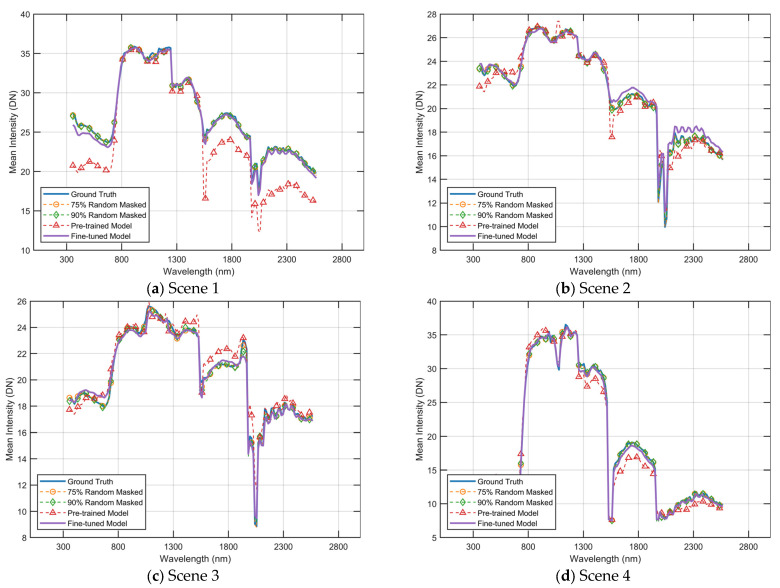
Reconstructed spectral curves comparisons of scenes shown in Figure 9 on the GF2Hyper dataset. The spectral curves were averaged in the spatial dimension of the images. DN is a digital number (uncalibrated) between 0 and 255.

**Figure 12:**
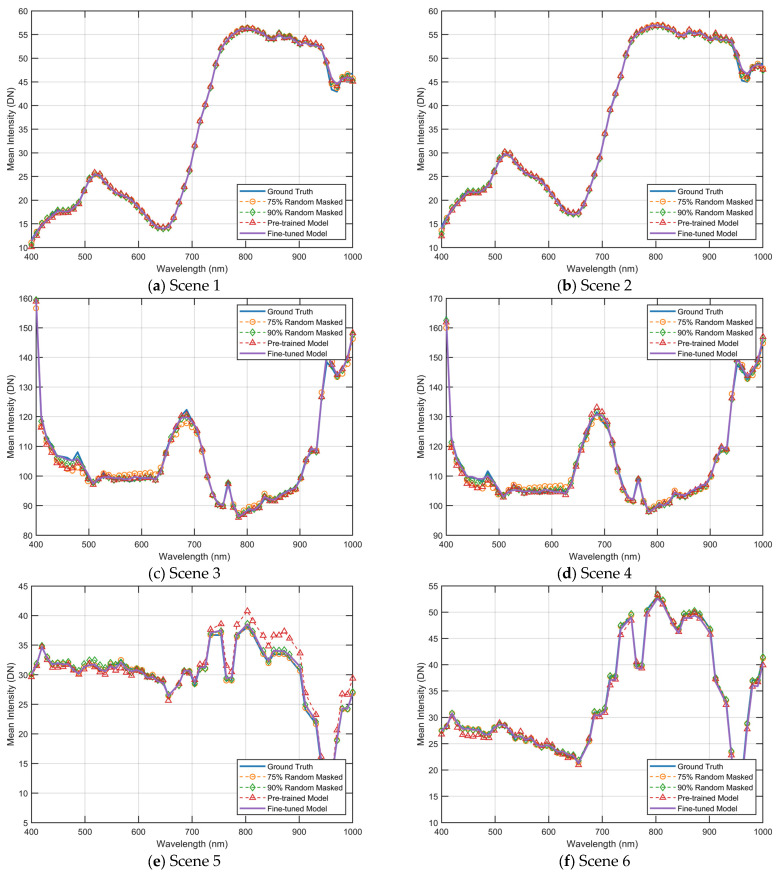
Reconstructed spectral curves comparisons of scenes shown in Figure 10. The spectral curves were averaged in the spatial dimension of the images. DN is a digital number (uncalibrated) between 0 and 255.

This section visually represents the reconstructed results under different strategies by comparing the spectral curves. As depicted in Figure 11 and Figure 12, the yellow curve illustrates the reconstruction outcome when utilizing a 75% masking rate and a pre-trained model with a 75% masking rate. The green curve illustrates the reconstruction outcome when utilizing a 90% masking rate, and the model is a pre-trained model that was trained with a 90% masking rate. The red curve represents the reconstruction results obtained by utilizing a fixed masking strategy, and the model used is a pre-trained model that was trained with a 90% masking rate. Furthermore, the purple curve represents the reconstruction results of a fine-tuned model that was obtained using the fixed masking strategy. It should be noted that the input for the reconstruction under the fixed masking strategy is limited to three-channel images.

As depicted in Figure 11, the results of the spectral curve reconstruction for the four scenes in Figure 9 are presented. It is evident that the reconstructed curves produced by the untuned pre-trained model deviate significantly from the true results.

As illustrated in Figure 12, the results of the spectral curve reconstruction for the six scenes in Figure 9 are presented. With regard to the Chikusei dataset, it is observed that the errors are concentrated in the wavelengths from 900 nm to 1000 nm, where the pre-trained model exhibits superior performance compared to the fine-tuned results. However, when considering the overall cumulative error results of the curves, the fine-tuned model demonstrates superior performance compared to the pre-trained model. For the XiongAn dataset, it is noted that the errors are primarily located within the wavelengths from 400 nm to 500 nm, where the fine-tuned model exhibits the best performance. Additionally, for the Washington DC dataset, it is observed that the errors are concentrated in wavelengths from 800 nm to 900 nm, where the values fluctuate significantly, and the fine-tuning significantly improves the results.

## 4. Conclusions

This paper proposed a spectral masked autoencoder network called SpectralMAE to deal with image reconstruction problems of hyperspectral remote sensing. Previous researchers have recently focused on developing supervised learning algorithms to address spectral reconstruction and super-resolution problems. The SpectralMAE method uses a self-supervised learning strategy that allows the model to acquire a holistic understanding beyond low-level spectral and spatial statistics. This self-supervised strategy is built on the spectral random masking mechanism proposed in this paper, and the spectral reconstruction effect with a 90% random masking rate is tested in experiments. The spectral random masking mechanism also enables the model to constitute a window-based spectral-wised attention, which can significantly reduce the model parameters compared to the imageMAE model.

Based on a random masking strategy, SpectralMAE can acquire pre-trained models for any combination of spectra. SpectralMAE can further train fine-tuned models to adapt to specific tasks, such as spectral reconstruction based on RGB images, using a fixed mask strategy. The study demonstrated the effectiveness of this strategy through comparative trials on multiple datasets.

The study was carried out in five public datasets of spectral images, including airborne and satellite remote sensing images. The study compared the HyRANK remote sensing spectral image dataset with 10 current state-of-the-art spectral reconstruction algorithms and super-resolution algorithms. It achieved optimal results in mPSNR, mSSIM, RMSE and SAM metrics. In the future, we will further improve our approach to the problem of spectral reconstruction after fusion between different sensors. We will further consider the integration between spatial blocks in the next study.

## Figures and Tables

**Table 1:** An overview of the state-of-the-art methods used for comparing the efficiency of proposed model.

Category	Algorithms	Basic Idea	Published Year
Transformer Network	MST++	Multi-stage Spectral-wise Transformer	2022
Transformer Network	MST	Mask-guided Spectral-wise Transformer	2022
Transformer Network	Restormer	Efficient Transformer model by making key designs in building blocks	2022
Attention Network	MPRNet	Multi-stage architecture for progressively learning restoration functions	2021
Attention Network	HRNet	Hierarchical regression network with PixelShuffle for inter-level interaction	2020
Attention Network	AWAN	Adaptive Weighted Attention Network with Camera Spectral Sensitivity Prior	2020
Residual Network	HINet	Applying Instance Normalization for half of the intermediate features while keeping content information	2022
Frequency domain learning	HDNet	HR pixel-level attention and frequency-level refinement for improved HSI perceptual quality	2022
Residual Network	EDSR	Enhanced super-resolution model with conventional ResNet architecture	2021
Dense Network	HSCNN+	Combination of two types of dense networks for restoration	2018

**Table 2:** Quantitative results on the HyRANK dataset.

Algorithms	mPSNR	mSSIM	RMSE	SAM
SpectralMAE	42.7851	0.97845	0.0138	3.7224
MST	42.2199	0.9674	0.024	5.9796
HINet	41.9321	0.9614	0.0199	5.9317
HRNet	40.6077	0.9592	0.0261	6.4218
Restormer	40.6778	0.9567	0.0249	6.5575
AWAN	40.3903	0.9508	0.035	7.9158
HSCNN+	41.0031	0.9465	0.0366	8.6661
EDSR	39.3331	0.9486	0.0316	7.6933
MST++	39.9776	0.9487	0.0267	7.7082
HDNet	38.7804	0.9516	0.0318	7.5014
MPRNet	38.2581	0.9352	0.0353	9.8121

**Table 3:** Quantitative results of pre-trained model by random masking strategy with 75% masking rate. The model is trained with a 75% masking rate.

Datasets	mPSNR	mSSIM	RMSE	SAM
Chikusei	47.9168	0.9927	0.0043	1.5218
Washington DC Mall	49.441	0.9963	0.0052	1.8181
XiongAn	41.5969	0.9921	0.0091	1.0483
GF2Hyper	46.4927	0.9981	0.0084	1.8812
HyRANK	44.1881	0.9855	0.0080	3.3170

**Table 4:** Quantitative results of pre-trained model by random masking strategy with 90% masking rate. The model is trained with a 90% masking rate.

Datasets	mPSNR	mSSIM	RMSE	SAM
Chikusei	43.3003	0.9843	0.0073	2.0728
Washington DC Mall	44.3279	0.9908	0.0090	2.6378
XiongAn	38.7479	0.9866	0.0121	1.1080
GF2Hyper	43.6433	0.9962	0.0100	2.5798
HyRANK	42.4905	0.9758	0.0101	4.3374

**Table 5:** Quantitative results of pre-trained model by fixed masking strategy with three channel inputs. The model is trained with a 90% masking rate.

Datasets	mPSNR	mSSIM	RMSE	SAM
Chikusei	44.3012	0.9858	0.0073	2.2141
Washington DC Mall	38.8888	0.9100	0.0306	10.7018
XiongAn	40.4450	0.9923	0.0111	1.17864
GF2Hyper	38.3501	0.9789	0.0150	5.8868
HyRANK	37.9810	0.9120	0.0332	12.2450

**Table 6:** Quantitative results of fine-tuned model by fixed masking strategy with three channel inputs. The model is trained with three channel inputs.

Datasets	mPSNR	mSSIM	RMSE	SAM
Chikusei	45.6253	0.9891	0.0066	2.0460
Washington DC Mall	44.6953	0.9737	0.0161	4.6298
XiongAn	41.1392	0.9932	0.0103	1.0755
GF2Hyper	42.0296	0.9932	0.0115	3.2380
HyRANK	42.7851	0.9784	0.0138	3.7224

## Data Availability

The HyRANK dataset is available at https://www2.isprs.org/commissions/comm3/wg4/hyrank/, accessed on 3 December 2022. The Sen2CHRIS dataset is available at https://zenodo.org/record/5808324, accessed on 3 December 2022. The GF2Hyper dataset is available at https://zenodo.org/record/5801045, accessed on 3 December 2022.

## References

[1] Tarabalka Y., Chanussot J., Benediktsson J.A. (2010). Segmentation and Classification of Hyperspectral Images Using Watershed Transformation. Pattern Recognit..

[2] Li J., Bioucas-Dias J.M., Plaza A. (2010). Semisupervised Hyperspectral Image Segmentation Using Multinomial Logistic Regression With Active Learning. IEEE Trans. Geosci. Remote Sens..

[3] Harsanyi J.C., Chang C.-I. (1994). Hyperspectral Image Classification and Dimensionality Reduction: An Orthogonal Subspace Projection Approach. IEEE Trans. Geosci. Remote Sens..

[4] Melgani F., Bruzzone L. (2004). Classification of Hyperspectral Remote Sensing Images with Support Vector Machines. IEEE Trans. Geosci. Remote Sens..

[5] Fauvel M., Benediktsson J.A., Chanussot J., Sveinsson J.R. (2008). Spectral and Spatial Classification of Hyperspectral Data Using SVMs and Morphological Profiles. IEEE Trans. Geosci. Remote Sens..

[6] Ren H., Chang C.-I. (2003). Automatic Spectral Target Recognition in Hyperspectral Imagery. IEEE Trans. Aerosp. Electron. Syst..

[7] Manolakis D., Shaw G. (2002). Detection Algorithms for Hyperspectral Imaging Applications. IEEE Signal. Process. Mag..

[8] Nguyen H.V., Banerjee A., Chellappa R. Tracking via Object Reflectance Using a Hyperspectral Video Camera. Proceedings of the 2010 IEEE Computer Society Conference on Computer Vision and Pattern Recognition—Workshops.

[9] Wang T., Zhu Z., Blasch E. (2010). Bio-Inspired Adaptive Hyperspectral Imaging for Real-Time Target Tracking. IEEE Sens. J..

[10] Jiang J., Sun H., Liu X., Ma J. (2020). Learning Spatial-Spectral Prior for Super-Resolution of Hyperspectral Imagery. IEEE Trans. Comput. Imaging.

[11] Yi C., Zhao Y.-Q., Chan J.C.-W. (2019). Spectral Super-Resolution for Multispectral Image Based on Spectral Improvement Strategy and Spatial Preservation Strategy. IEEE Trans. Geosci. Remote Sens..

[12] Hu X., Cai Y., Lin J., Wang H., Yuan X., Zhang Y., Timofte R., Van Gool L. HDNet: High-Resolution Dual-Domain Learning for Spectral Compressive Imaging. Proceedings of the 2022 IEEE/CVF Conference on Computer Vision and Pattern Recognition (CVPR).

[13] Yuan Y., Zheng X., Lu X. (2017). Hyperspectral Image Superresolution by Transfer Learning. IEEE J. Sel. Top. Appl. Earth Obs. Remote Sens..

[14] Koundinya S., Sharma H., Sharma M., Upadhyay A., Manekar R., Mukhopadhyay R., Karmakar A., Chaudhury S. 2D-3D CNN Based Architectures for Spectral Reconstruction from RGB Images. Proceedings of the 2018 IEEE/CVF Conference on Computer Vision and Pattern Recognition Workshops (CVPRW).

[15] Li J., Wu C., Song R., Li Y., Liu F. Adaptive Weighted Attention Network with Camera Spectral Sensitivity Prior for Spectral Reconstruction from RGB Images. Proceedings of the 2020 IEEE/CVF Conference on Computer Vision and Pattern Recognition Workshops (CVPRW).

[16] Liu P., Zhao H. (2020). Adversarial Networks for Scale Feature-Attention Spectral Image Reconstruction from a Single RGB. Sensors.

[17] Shi Z., Chen C., Xiong Z., Liu D., Wu F. HSCNN+: Advanced CNN-Based Hyperspectral Recovery from RGB Images. Proceedings of the 2018 IEEE/CVF Conference on Computer Vision and Pattern Recognition Workshops (CVPRW).

[18] Cai Y., Lin J., Lin Z., Wang H., Zhang Y., Pfister H., Timofte R., Gool L.V. MST++: Multi-Stage Spectral-Wise Transformer for Efficient Spectral Reconstruction. Proceedings of the 2022 IEEE/CVF Conference on Computer Vision and Pattern Recognition Workshops (CVPRW).

[19] Zhang J., Su R., Fu Q., Ren W., Heide F., Nie Y. (2021). A Survey on Computational Spectral Reconstruction Methods from RGB to Hyperspectral Imaging. Sci. Rep..

[20] Wu J., Aeschbacher J., Timofte R. In Defense of Shallow Learned Spectral Reconstruction from RGB Images. Proceedings of the 2017 IEEE International Conference on Computer Vision Workshops (ICCVW).

[21] Arad B., Ben-Shahar O., Leibe B., Matas J., Sebe N., Welling M. (2016). Sparse Recovery of Hyperspectral Signal from Natural RGB Images. Computer Vision—ECCV 2016.

[22] Li Y., Wang C., Zhao J. (2018). Locally Linear Embedded Sparse Coding for Spectral Reconstruction From RGB Images. IEEE Signal. Process. Lett..

[23] Fu Y., Zheng Y., Zhang L., Huang H. (2018). Spectral Reflectance Recovery From a Single RGB Image. IEEE Trans. Comput. Imaging.

[24] Geng Y., Mei S., Tian J., Zhang Y., Du Q. Spatial Constrained Hyperspectral Reconstruction from RGB Inputs Using Dictionary Representation. Proceedings of the IGARSS 2019—2019 IEEE International Geoscience and Remote Sensing Symposium.

[25] Akhtar N., Mian A. (2020). Hyperspectral Recovery from RGB Images Using Gaussian Processes. IEEE Trans. Pattern Anal. Mach. Intell..

[26] Jia Y., Zheng Y., Gu L., Subpa-Asa A., Lam A., Sato Y., Sato I. From RGB to Spectrum for Natural Scenes via Manifold-Based Mapping. Proceedings of the 2017 IEEE International Conference on Computer Vision (ICCV).

[27] Xiong Z., Shi Z., Li H., Wang L., Liu D., Wu F. HSCNN: CNN-Based Hyperspectral Image Recovery from Spectrally Undersampled Projections. Proceedings of the 2017 IEEE International Conference on Computer Vision Workshops (ICCVW).

[28] Alvarez-Gila A., Van De Weijer J., Garrote E. Adversarial Networks for Spatial Context-Aware Spectral Image Reconstruction from RGB. Proceedings of the 2017 IEEE International Conference on Computer Vision Workshops (ICCVW).

[29] Wang B., Zhu L., Guo X., Wang X., Wu J. (2022). SDTGAN: Generation Adversarial Network for Spectral Domain Translation of Remote Sensing Images of the Earth Background Based on Shared Latent Domain. Remote Sens..

[30] He J., Yuan Q., Li J., Xiao Y., Liu X., Zou Y. (2022). DsTer: A Dense Spectral Transformer for Remote Sensing Spectral Super-Resolution. Int. J. Appl. Earth Obs. Geoinf..

[31] Cai Y., Lin J., Hu X., Wang H., Yuan X., Zhang Y., Timofte R., Van Gool L. Mask-Guided Spectral-Wise Transformer for Efficient Hyperspectral Image Reconstruction. Proceedings of the 2022 IEEE/CVF Conference on Computer Vision and Pattern Recognition (CVPR).

[32] Stiebei T., Koppers S., Seltsam P., Merhof D. Reconstructing Spectral Images from RGB-Images Using a Convolutional Neural Network. Proceedings of the 2018 IEEE/CVF Conference on Computer Vision and Pattern Recognition Workshops (CVPRW).

[33] Kaya B., Can Y.B., Timofte R. Towards Spectral Estimation from a Single RGB Image in the Wild. Proceedings of the 2019 IEEE/CVF International Conference on Computer Vision Workshop (ICCVW).

[34] Zhang L., Lang Z., Wang P., Wei W., Liao S., Shao L., Zhang Y. Pixel-Aware Deep Function-Mixture Network for Spectral Super-Resolution. Proceedings of the AAAI Conference on Artificial Intelligence.

[35] Liu X., Zhang F., Hou Z., Mian L., Wang Z., Zhang J., Tang J. (2021). Self-Supervised Learning: Generative or Contrastive. IEEE Trans. Knowl. Data Eng..

[36] He K., Chen X., Xie S., Li Y., Dollar P., Girshick R. Masked Autoencoders Are Scalable Vision Learners. Proceedings of the 2022 IEEE/CVF Conference on Computer Vision and Pattern Recognition (CVPR).

[37] Dosovitskiy A., Beyer L., Kolesnikov A., Weissenborn D., Zhai X., Unterthiner T., Dehghani M., Minderer M., Heigold G., Gelly S. (2021). An Image Is Worth 16x16 Words: Transformers for Image Recognition at Scale. arXiv.

[38] Vaswani A., Shazeer N., Parmar N., Uszkoreit J., Jones L., Gomez A.N., Kaiser L., Polosukhin I. Attention Is All You Need. Proceedings of the 31st International Conference on Neural Information Processing Systems.

[39] Zhao H., Jia J., Koltun V. Exploring Self-Attention for Image Recognition. Proceedings of the 2020 IEEE/CVF Conference on Computer Vision and Pattern Recognition (CVPR).

[40] Liu Z., Lin Y., Cao Y., Hu H., Wei Y., Zhang Z., Lin S., Guo B. Swin Transformer: Hierarchical Vision Transformer Using Shifted Windows. Proceedings of the 2021 IEEE/CVF International Conference on Computer Vision (ICCV).

[41] Zhao Y., Po L.-M., Yan Q., Liu W., Lin T. Hierarchical Regression Network for Spectral Reconstruction from RGB Images. Proceedings of the 2020 IEEE/CVF Conference on Computer Vision and Pattern Recognition Workshops (CVPRW).

[42] Zamir S.W., Arora A., Khan S., Hayat M., Khan F.S., Yang M.-H., Shao L. Multi-Stage Progressive Image Restoration. Proceedings of the 2021 IEEE/CVF Conference on Computer Vision and Pattern Recognition (CVPR).

[43] Zamir S.W., Arora A., Khan S.H., Hayat M., Khan F.S., Yang M.-H. Restormer: Efficient Transformer for High-Resolution Image Restoration. Proceedings of the 2022 IEEE/CVF Conference on Computer Vision and Pattern Recognition (CVPR).

[44] Chen L., Lu X., Zhang J., Chu X., Chen C. HINet: Half Instance Normalization Network for Image Restoration. Proceedings of the 2021 IEEE/CVF Conference on Computer Vision and Pattern Recognition Workshops (CVPRW).

[45] Lim B., Son S., Kim H., Nah S., Lee K.M. Enhanced Deep Residual Networks for Single Image Super-Resolution. Proceedings of the 2017 IEEE Conference on Computer Vision and Pattern Recognition Workshops (CVPRW).

[46] Arad B., Timofte R., Yahel R., Morag N., Bernat A., Cai Y., Lin J., Lin Z., Wang H., Zhang Y. NTIRE 2022 Spectral Recovery Challenge and Data Set. Proceedings of the 2022 IEEE/CVF Conference on Computer Vision and Pattern Recognition Workshops (CVPRW).

[47] Arad B., Liu D., Wu F., Lanaras C., Galliani S., Schindler K., Stiebel T., Koppers S., Seltsam P., Zhou R. NTIRE 2018 Challenge on Spectral Reconstruction from RGB Images. Proceedings of the 2018 IEEE/CVF Conference on Computer Vision and Pattern Recognition Workshops (CVPRW).

[48] Arad B., Timofte R., Ben-Shahar O., Lin Y.-T., Finlayson G., Givati S., Li J., Wu C., Song R., Li Y. NTIRE 2020 Challenge on Spectral Reconstruction from an RGB Image. Proceedings of the 2020 IEEE/CVF Conference on Computer Vision and Pattern Recognition Workshops (CVPRW).

[49] Yokoya N., Iwasaki A. Airborne Unmixing-Based Hyperspectral Super-Resolution Using RGB Imagery. Proceedings of the 2014 IEEE Geoscience and Remote Sensing Symposium.

[50] Biehl L., Landgrebe D. (2002). MultiSpec—A Tool for Multispectral–Hyperspectral Image Data Analysis. Comput. Geosci..

[51] Cen Y., Zhang L., Zhang X., Wang Y., Qi W., Tang S., Zhang P. (2020). Aerial Hyperspectral Remote Sensing Classification Dataset of Xiongan New Area (Matiwan Village). J. Remote Sens..

[52] Meng Z., Zhao F., Liang M., Xie W. (2021). Deep Residual Involution Network for Hyperspectral Image Classification. Remote Sens..

[53] He J., Yuan Q., Li J., Zhang L. (2022). PoNet: A Universal Physical Optimization-Based Spectral Super-Resolution Network for Arbitrary Multispectral Images. Inf. Fusion..

[54] Wang Z., Bovik A.C., Sheikh H.R., Simoncelli E.P. (2004). Image Quality Assessment: From Error Visibility to Structural Similarity. IEEE Trans. Image Process..

[55] Kruse F.A., Lefkoff A.B., Boardman J.W., Heidebrecht K.B., Shapiro A.T., Barloon P.J., Goetz A.F.H. (1993). The Spectral Image Processing System (SIPS)—Interactive Visualization and Analysis of Imaging Spectrometer Data. Remote Sens. Environ..

